# Dissociation of category-learning systems via brain potentials

**DOI:** 10.3389/fnhum.2015.00389

**Published:** 2015-07-07

**Authors:** Robert G. Morrison, Paul J. Reber, Krishna L. Bharani, Ken A. Paller

**Affiliations:** ^1^Department of Psychology, Neuroscience Institute, Loyola University ChicagoChicago, IL, USA; ^2^Department of Psychology, Northwestern UniversityEvanston, IL, USA; ^3^Department of Neuroscience, Medical University of South CarolinaCharleston, SC, USA

**Keywords:** category learning, event-related potentials, explicit, implicit, EEG

## Abstract

Behavioral, neuropsychological, and neuroimaging evidence has suggested that categories can often be learned via either an explicit rule-based (RB) mechanism critically dependent on medial temporal and prefrontal brain regions, or via an implicit information-integration (II) mechanism relying on the basal ganglia. In this study, participants viewed sine-wave gratings (Gabor patches) that varied on two dimensions and learned to categorize them via trial-by-trial feedback. Two different stimulus distributions were used; one was intended to encourage an explicit RB process and the other an implicit II process. We monitored brain activity with scalp electroencephalography (EEG) while each participant: (1) passively observed stimuli represented of both distributions; (2) categorized stimuli from one distribution, and, 1 week later; (3) categorized stimuli from the other distribution. Categorization accuracy was similar for the two distributions. Subtractions of Event-Related Potentials (ERPs) for correct and incorrect trials were used to identify neural differences in RB and II categorization processes. We identified an occipital brain potential that was differentially modulated by categorization condition accuracy at an early latency (150–250 ms), likely reflecting the degree of holistic processing. A stimulus-locked Late Positive Complex (LPC) associated with explicit memory updating was modulated by accuracy in the RB, but not the II task. Likewise, a feedback-locked P300 ERP associated with expectancy was correlated with performance only in the RB, but not the II condition. These results provide additional evidence for distinct brain mechanisms supporting RB vs. implicit II category learning and use.

## Introduction

Categories, as conceptualized based on perceived regularities, allow us to make sense of, describe, and order our worlds (Rips et al., 2012). Categories come in many different forms—from categories based on a single feature (e.g., objects that are red) to much more complicated relational concepts (e.g., *chases* or *conduit*). Many have argued that human categorization is not a unitary process, but rather can engage different systems depending on the category structure or the conditions during category learning (e.g., Yamauchi and Markman, 1998; Nomura and Reber, 2008; Smith and Grossman, 2008; Seger and Miller, 2010; Ashby and Maddox, 2011). Behavioral, neuropsychological, and neuroimaging evidence suggests that these various systems can make differential demands on neural networks of the brain (e.g., Kéri, 2003; Nomura and Reber, 2008; Smith and Grossman, 2008; Seger and Miller, 2010; Ashby and Maddox, 2011). However, describing the algorithm and neural implementation of category-learning systems, as well as the factors that determine when each system will be engaged and how these systems interact, is still a very active endeavor.

**Figure 1:**
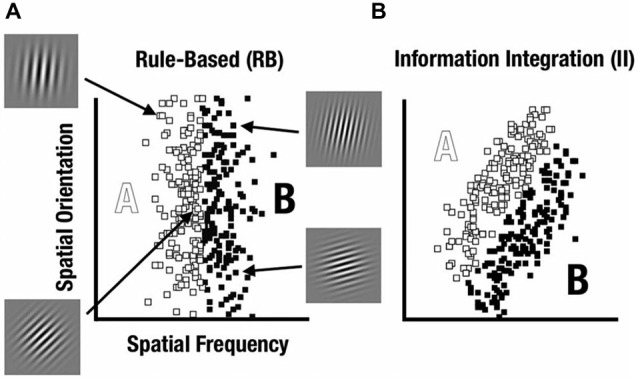
**Rule-based (RB) and information-integration (II) category distributions used in the study**. Sine-wave gratings varied based on spatial frequency and spatial orientation. **(A)** The RB category was defined based on frequency whereas orientation varied unsystematically. **(B)** The II category was defined based on both frequency and orientation with a diagonal decision bound.

A prominent way to characterize category-learning systems postulates distinct rule-based (RB) and information-integration (II) categorization strategies that engage different neurocognitive networks (see Ashby and Maddox, 2011). Within this framework, Maddox et al. (2003) have developed a feedback category-learning paradigm which parametrically varies the perceptual properties of sine-wave gratings (Gabor patches) to create category distributions that encourage either RB or II category learning strategies (see Figure 1).

RB tasks are those where the categories can be learned via a reasoning process such as hypothesis testing (Ashby et al., 1998, 2005). In contrast, II category learning and use appears to occur implicitly, such that the rule for the category structure is difficult to learn consciously or to describe verbally. After learning, participants can explicitly describe the rule they use to categorize the stimuli. This RB mechanism would require maintaining and updating the rule and the boundary condition, requiring the use of both working memory, dependent on prefrontal cortex (PFC), and long-term memory, dependent on medial temporal lobe (MTL; Nomura and Reber, 2012).

In contrast, II learning appears to occur implicitly, such that the rule for the category structure is difficult to learn consciously or to describe verbally. II tasks appear to encourage participants to consider the stimuli holistically, integrating perceptual information from different stimulus features early during processing. II learning may depend on implicit learning supported by computations involving the caudate nucleus and visual processing areas in occipital cortex (Nomura and Reber, 2012). Dopaminergic reward circuits of the caudate may be responsible for associating specific categories with neuronal patterns in occipital cortex that code for relevant visual features (Ashby et al., 1998).

Numerous behavioral experiments comparing RB and II category learning have shown that they are employed using dissociable strategies. For example, working memory dual-task procedures interfered with RB much more than with II learning (e.g., Zeithamova and Maddox, 2006, 2007). Delaying feedback beyond an initial period did not interfere with RB learning but disrupted II learning (e.g., Maddox et al., 2003). Changing the response key associated with a particular category also interfered with II but not RB categorization, suggesting that II learning may require stimulus-response association learning with relatively immediate feedback, characteristics associated with implicit procedural learning (Ashby et al., 2003).

Mechanistically RB processing is thought to depend on hypothesis testing. For instance a participant trying to categorize line segments into two groups might hypothesize that length is what matters, with long segments being one category and short segments being the other. On each trial they test their theory with a response to each line segment. While they may find support for their theory quickly they gradually build a representation of the category threshold that allows them to improve their performance. After each test of their hypothesis they then need to update their memory with whether the test worked and with a candidate threshold value. This evaluation requires selective attention and working memory, likely implemented in PFC, as well as the ability to form enduring mental representations of the rule and boundary condition dependent on the hippocampus and MTL. In contrast, II learning is believed to require information integration of multiple stimulus attributes at a predecisional stage (Ashby et al., 1998). Unlike in RB learning, learners frequently cannot articulate what they have learned, but can show their learning through successful performance, a hallmark of nondeclarative memory (Squire, 2009). Thus, II learning may be likened to gaining category expertize with complex objects such as faces (Bentin et al., 1996) or Greebles (Rossion et al., 2002).

Working from this distinction, functional magnetic resonance imaging (fMRI) methods have been useful to spatially dissociate the brain networks responsible for categorization and use when participants learn either an RB or II category distribution. In a study by Nomura et al. (2007b), participants who learned the RB distribution showed greater activation in the MTL on correct than incorrect trials, while participants who learned the II distribution showed greater activation in the body of the caudate on correct than incorrect trials. Another category learning study using a different paradigm likewise found activity in the body and tail of the caudate and putamen to be active when learning stimulus-category associations (Cincotta and Seger, 2007). Nomura and Reber (2012) subsequently reanalyzed several sets of RB/II paradigm fMRI data (Nomura et al., 2007a) using PINNACLE (Parallel Interactive Neural Networks Active in Category Learning), a computational model that includes multiple competing categorization systems. Using a participant’s behavioral decision data, PINNACLE employs principals of Decision-Bound Modeling Theory (Ashby and Maddox, 1993) to estimate which categorization system is likely engaged on a given trial. Thus, PINNACLE can be used to sort trials of neuroimaging data to obtain estimates of the neural correlates for individual category-learning systems. This approach identified areas in PFC important for correct decisions during RB category learning, a finding consistent with another previous fMRI study of RB category learning (Filoteo et al., 2005). Posterior regions of occipital cortex were associated with correct decisions during II category learning, a finding consistent with previous fMRI studies of implicit category learning (Reber et al., 1998a,b; Waldschmidt and Ashby, 2011). In addition, this approach found evidence that regions of dorsolateral PFC were involved in the process of resolving competition between the two systems based on the model-identified moments of high levels of inter-system competition.

Further progress in understanding the neurocognitive mechanisms of category learning will depend on the ability to measure relevant processing. In particular, measures with high temporal resolution are needed to comprehensively distinguish RB and II mechanisms. In the present study we computed event-related potentials (ERPs) from scalp electroencephalographic (EEG) recordings to examine neural correlates of category learning during both categorization and feedback stages. Participants learned RB and II category distributions during separate testing sessions and their responses were analyzed using Decision-Bound Modeling Theory (Ashby and Maddox, 1993) to identify participants likely to be using RB and II category learning processes with corresponding distributions. Based on prior behavioral and neuroimaging results, we anticipated that RB and II category learning mechanisms would produce different ERPs, when comparing successful (correct) and unsuccessful (incorrect) trials. Specifically, we anticipated differences in an early occipital N1 ERP previously associated with visual category learning (Curran et al., 2002), and consistent with occipital activation found for II category learning in our previous work (Nomura and Reber, 2012). Secondly, given the previously demonstrated reliance of RB category learning on MTL (Seger and Cincotta, 2006; Nomura et al., 2007a; Seger et al., 2011) we predicted that a Late Positive Complex (LPC) ERP associated with explicit memory (Voss and Paller, 2008) would be modulated by accuracy in the RB condition but not the II condition. Lastly, to the extent that RB learning is more explicit than II learning (Huang-Pollock et al., 2011; Seger et al., 2011), we anticipated that the P300 to positive feedback would index participant’s confidence in their learning (Hajcak et al., 2005).

## Materials and Methods

### Task Description

**Figure 2:**
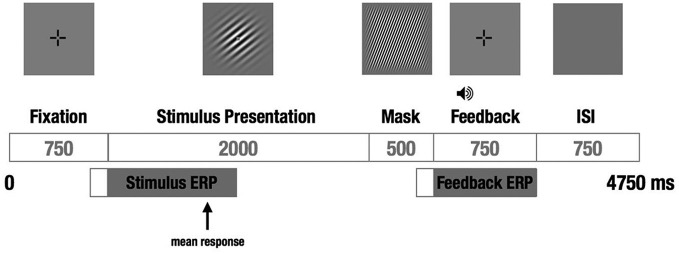
**Schematic of a single trial**. A fixation cross was followed by the to-be-categorized-stimulus for a fixed duration, followed by a short visual mask, followed by auditory feedback and a brief ISI before the next trial. The subject responded “category A” or “category B” during the 2 s the stimulus was on the screen by pressing one of two buttons on a hand-held response box. EEG was recorded continuously, and stimulus- and feedback-locked ERPs were calculated from each trial.

We used a visual category-learning paradigm (Maddox et al., 2003) in which subjects learned to categorize visual stimuli into two categories via feedback given at the conclusion of each trial. Stimuli were circular sine-wave gratings that varied in spatial frequency (number of lines per patch, also perceived as thickness of lines) and spatial orientation (tilt of lines). For the RB distribution, the stimuli were divided into two categories based on a vertical decision boundary such that category membership depended only on the spatial frequency of the sine-wave grating (Figure 1A). For the II group, the categories were defined by a diagonal decision boundary that required II of frequency and orientation information (Figure 1B). Trial timing was similar to that used by Nomura et al. (2007a) in their fMRI study (Figure 2).

### Participants

Twenty-eight Northwestern University students served as participants in this experiment. Participants received US$15 per hour for two 2 to 3 hr testing sessions. Participants categorized the RB and II category distributions in separate sessions 1 week apart. Distribution order was counterbalanced across participants. Participants gave informed consent according to the oversight of the Northwestern University Institutional Review Board.

### Procedure

#### Prelearning

In order to rule out differences in ERPs due to differences in the physical stimuli in the RB and II distributions, participants passively viewed 160 sine-wave gratings from both distributions over the course of two blocks prior to attempting to learn categories. Gratings were representative of the range of spatial frequency and orientation used during category learning. During prelearning participants received no instruction that categories of stimuli were present or that they should categorize. Prelearning trial timing was identical to that during category learning, but participants did not make a response during prelearning and thus received no feedback.

#### Category Learning

Participants categorized 320 sine-wave gratings presented in four blocks during each category-learning session. One session involved the RB distribution and the other session involved the II distribution. Distribution order was counterbalanced across participants. Prior to testing, subjects were familiarized with the procedures, including trial timing, button pressing, and feedback. Participants did not receive instructions about the nature of the categories; rather, they were asked to discover the categories with the aid of auditory feedback. Participants received auditory feedback 2.5 s after stimulus onset. For a correct decision the feedback was a bell sound. For incorrect decisions the feedback was a short buzzer, while participants heard a long buzzer of equal duration when no response was made in the allotted 2 s. Responses after 2 s were not considered in the analysis. Subjects were debriefed about their categorization strategies after the second testing session.

#### EEG

**Figure 3:**
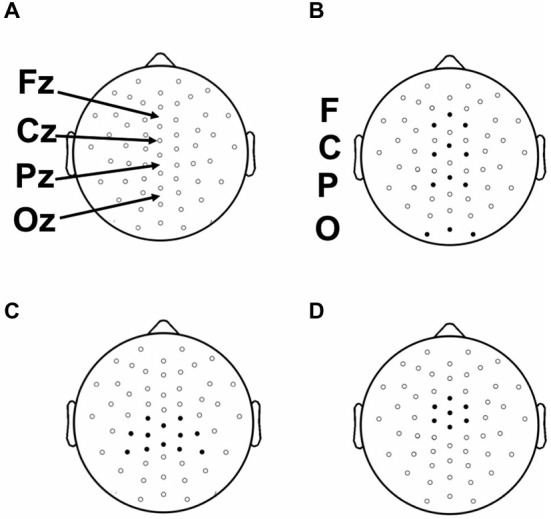
**(A)** Placement of 52 scalp electrodes (of 58 total) used in this study with respect to 10–20 landmarks. **(B)** Frontal (F), Central (C), Parietal (P), and Occipital (O) electrode clusters used for the analysis of the N1. **(C)** Parietal electrode cluster surrounding location Pz used in the analysis of the Late positive complex (LPC). **(D)** Central electrode cluster surrounding and including electrode Cz used in the analysis of the feedback P300.

Continuous EEG recordings were made during prelearning and category-learning blocks from 59 evenly distributed scalp sites using tin electrodes embedded in an elastic cap (Figure 3). Four additional electrodes were used for monitoring horizontal and vertical eye movements, and two electrodes were placed over the left and right mastoid bones. Participants were instructed to attempt to refrain from blinking or moving their eye position from fixation during the categorization and feedback portions of each trial. Electrode impedance was ≤5 kΩ. EEG signals were amplified with a band pass of 0.05–200 Hz and sampled at a rate of 1000 Hz. The online reference (left mastoid) was changed to average mastoids offline and a 59 to 60 Hz band-stop filter was applied. EMSE Software Suite (Source Signal Imaging, San Diego, CA, USA) was used to process raw EEG files and to compute ERPs. Electrooculograph (EOG) artifacts were corrected by using a blink-correction algorithm based on independent component analysis. Averaging epochs for stimulus and feedback lasted 1200 ms, including a 200 ms pre-stimulus baseline. Trials showing a greater than 100 μV deflection during the epoch were discarded. Fewer than 15% of trials were excluded for any given condition for any given participant.

#### Decision-Bound Theory Modeling

Although participants received stimuli drawn from either the RB distribution or from the II distribution within each session, some participants would be expected to fail to adopt the optimal categorization strategy. As in prior work (Ashby and Maddox, 1993; Nomura and Reber, 2012), we used Decision-Bound Theory (DBT) models to classify behavioral patterns as consistent with either an RB strategy or II strategy. For each participant, the pattern of categorization responses across the stimulus space was compared to an RB-F model based on stimulus spatial frequency (thinness of the black/white strips reflected as a vertical boundary in stimulus space), an RB-O model based on spatial orientation (angle of the black/white strips reflected as a horizontal boundary in stimulus space) and an II model based on a diagonal partition of the stimulus space. The specific placement of the category boundary was optimized to the participant’s behavior and the quality of the fit was contrasted across models. By this method, performance in each session can be identified as consistent with either an RB or II approach that either is relatively optimal for the administered stimulus set or reflects a suboptimal strategy. We fit each block of 80 trials using the DBT model. Participants whose performance was consistent with task demands (i.e., at least three of four blocks showed model-to-distribution agreement) were considered the Model-Conforming group and the remaining participants were designated as the Model-Nonconforming group. Using this technique to identify participants most clearly expressing the appropriate strategy strengthens the comparison of ERP differences between RB and II category learning.

## Results

**Figure 4:**
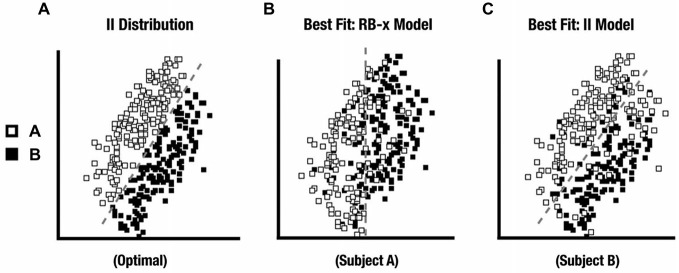
**(A)** II distribution used in the experiment. **(B)** II distribution category responses from a participant whose responses were best fit by a RB decision-bound theory (DBT) model and who was excluded from further analysis. **(C)** II distribution category responses from a participant whose responses were best fit by an II DBT model and who was kept for further analysis.

All 28 participants exhibited an RB distribution response best fit by an RB-F DBT model. For II, only 15 participants comprised the Model-Conforming Group because they exhibited an II distribution response profile best fit by an II DBT model. In contrast, 13 participants comprised the Model-Nonconforming Group because they exhibited an II distribution response profile best fit by an RB-F or RB-O DBT model (see Figure 4 for distribution profiles from representative participants). Likewise, when the fits for these two groups were compared directly, the first group of participants exhibited better II model fits than did the second (*t* (26) = 2.7, *p* = 0.01). However, these two groups did not differ in the quality of their RB model fits with the RD distribution (*t* (26) = 0.02, *ns*). DBT model fitting thus allowed data from participants who were likely using a unidimensional RB strategy with the II category distributions to be excluded from subsequent analyses.

### Behavioral Performance

Of the 15 participants whose DBT fits were consistent with II strategy use with II distributions, two did not have an adequate number of incorrect trials (<30) to allow for the correct/incorrect ERP analysis, so their results were excluded from further analysis. Data from one additional participant were eliminated because of poor EEG quality.

**Figure 5:**
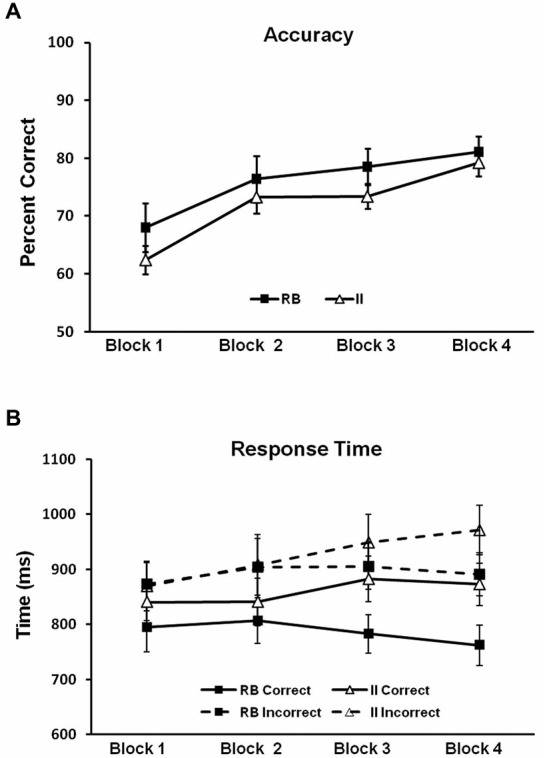
**Behavioral results for Model-Conforming Group. (A)** Accuracy and **(B)** RTs for participants included based on DBT model fits and included in the analysis of brain potentials. Error bars represent ±1 standard error of the mean.

To evaluate potential differences in category-learning accuracy for the RB and II distributions, we ran a 2 (RB vs. II) by 4 (block) repeated-measures ANOVA. Accuracy for RB and II distributions (Figure 5A) did not reliably differ (*F*_(1,11)_ = 1.6, *p* = 0.23, *η*_p_^2^ = 0.13). There was a main effect of block (*F*_(3,33)_ = 24, *p* < 0.001, *η*_p_^2^ = 0.69), and category learning linearly increased over blocks (*F*_(1,11)_ = 50, *p* < 0.001, *η*_p_^2^ = 0.81). However, RB and II distributions did not differ with respect to this pattern (*F*_(1,11)_ = 0.4, *p* = 0.5, *η*_p_^2^ = 0.04). Thus, observed differences in correct/incorrect ERP subtractions (described below) cannot easily be attributed to differences in accuracy between RB and II learning.

Next we looked for potential differences in category-learning RT for the RB and II distributions by using a 2 (RB vs. II) by 2 (Correct vs. Incorrect) by 4 (block) repeated measures ANOVA (see Figure 5B). Participants were faster on correct than incorrect trials (*F*_(1,11)_ = 27, *p* < 0.001, *η*_p_^2^ = 0.71). There was also a trend towards faster responses on RB trials compared to II trials (*F*_(1,11)_ = 4.0, *p* = 0.07, *η*_p_^2^ = 0.27). Likewise, there was a trend suggesting an interaction between accuracy and distribution type (*F*_(1,11)_ = 2.6, *p* = 0.14, *η*_p_^2^ = 0.19). Participants were faster on correct trials than on incorrect trials for both RB distributions (*F*_(1,11)_ = 20, *p* < 0.001, *η*_p_^2^ = 0.65) and II distributions (*F*_(1,11)_ = 14, *p* = 0.003, *η*_p_^2^ = 0.56). However, RB and II trials only differed for correct trials (*F*_(1,11)_ = 6.6, *p* = 0.026, *η*_p_^2^ = 0.38) not incorrect trials (*F*_(1,11)_ = 1.1, *p* = 0.31, *η*_p_^2^ = 0.09).

### EEG Results

#### Categorization ERPs

**Figure 6:**
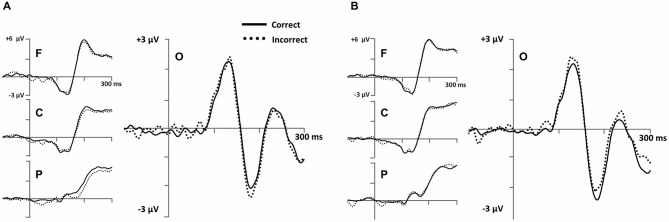
**Early stimulus-locked ERPs from frontal (F; three marked electrodes just posterior to Fz), central (C; three marked electrodes including Cz), parietal (P; three marked electrodes just posterior to Pz) and occipital (three marked electrodes just posterior to Oz including the Iniun) electrode clusters (see Figure 3B for precise electrode locations) for **(A)** RB and **(B)** II category-learning conditions**.

**Figure 7:**
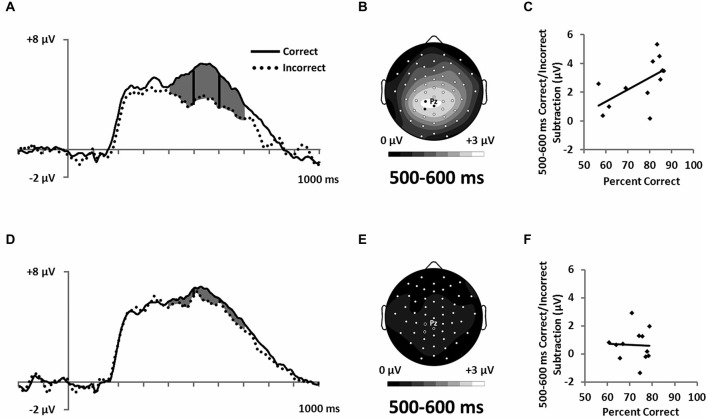
**ERPs showing the LPC ERP for both (A) RB and (D) II conditions in a cluster of parietal electrodes (12 marked electrodes surrounding Pz; see Figure 3C for precise electrode locations).** Topographic maps representing correct minus incorrect subtractions from 500–600 ms for **(B)** RB and **(E)** II ERPs. Scatterplots showing the relationship of accuracy to the correct minus incorrect mean amplitude ERP subtractions from 500–600 ms for three parietal electrodes near Pz (indicated on the corresponding topographic maps) for **(C)** RB and **(F)** II conditions.

Based on our predictions, stimulus-locked analyses were focused on an early occipital N1 ERP (Figure 6) and a later parietal LPC ERP (Figure 7) in the Model-Conforming Group.

To measure occipital N1 ERPs, we calculated mean amplitude from 150–250 ms for a cluster of inferior occipital electrodes (Figure 6). The same electrodes and time range were used for every participant. This time range included the occipital N1 peak for all participants. A 2 (RB vs. II) by 2 (Correct vs. Incorrect) ANOVA performed on mean amplitudes yielded a reliable interaction between distribution type and accuracy (*F*_(1,11)_ = 6.1, *p* = 0.03, *η*_p_^2^ = 0.36), but no main effect of distribution type (*F*_(1,11)_ = 0.05, *p* = 0.8, *η*_p_^2^ = 0.004) or accuracy (*F*_(1,11)_ = 0.04, *p* = 0.9, *η*_p_^2^ = 0.003). Amplitudes at this latency for correct and incorrect trials were reliably different for the II distribution (*F*_(1,11)_ = 6.3, *p* = 0.03, *η*_p_^2^ = 0.37) and showed a trend in the opposite direction for the RB distribution (*F*_(1,11)_ = 2.6, *p* = 0.14, *η*_p_^2^ = 0.19).

Also consistent with predictions, we found a stimulus-locked LPC ERP largest over the parietal electrodes (Figure 7A,D). To quantify LPC, we measured mean amplitude from 400–700 ms in a cluster of parietal electrodes (see Figure 3C). A 2 (RB vs. II) by 2 (Correct vs. Incorrect) ANOVA performed on mean amplitudes yielded a reliable interaction between distribution type and accuracy (*F*_(1,11)_ = 9.6, *p* = 0.01, *η*_p_^2^ = 0.47). The LPC was reliably larger for correct than incorrect trials in the RB condition (*F*_(1,11)_ = 20, *p* = 0.001, *η*_p_^2^ = 0.65), but not in the II condition (*F*_(1,11)_ = 3.2, *p* = 0.1, *η*_p_^2^ = 0.23). To uncover relationships between this ERP and performance (Figure 7B,D), we used a smaller parietal region and temporal window (500–600 ms) targeted for maximal mean amplitude differences as a function of accuracy. Magnitude of the Correct/Incorrect ERP differences were reliably correlated with RB performance (Figure 7C; *r*_(11)_ = 0.68, *p* = 0.01) but not with II performance (Figure 7F; *r*_(11)_ = 0.05, *p* = 0.9).

#### Feedback ERPs

**Figure 8:**
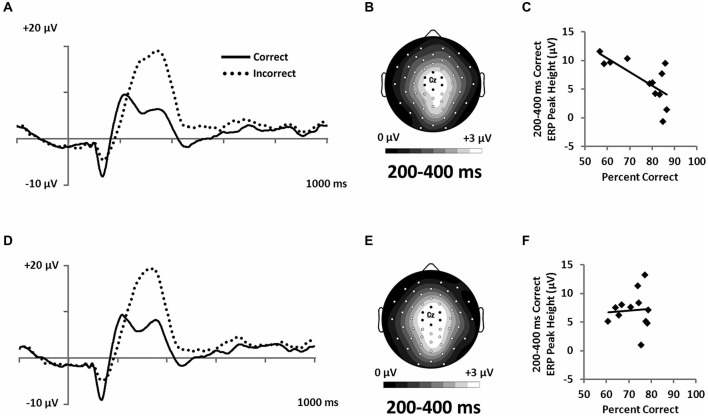
**Feedback-locked ERPs from a central cluster of electrodes (seven marked electrodes surrounding Cz; see Figure 3D for precise electrode locations) for (A) RB and (D) II category-learning conditions**. Topographic maps representing mean amplitude from correct responses from 200–400 ms for **(B)** RB and **(E)** II ERPs. Scatterplots showing the relationship of accuracy to mean amplitude for correct trials for **(C)** RB and **(F)** II conditions.

In order to assess hypotheses about the extent to which categorization was based on explicit knowledge, we examined ERPs recorded during feedback (Figure 8). Participants interpret feedback signals as a function of their explicit expectations. P300 responses have been associated with confidence in learning with feedback (Hajcak et al., 2005). Accordingly, we expected P300 potentials to index learning in the RB but not in the II condition, given that explicit learning mechanisms are thought to dominate in the RB but not the II condition. Both Correct and Incorrect trials showed large positive potentials at approximately 300 ms with central-focused topographies (Figures 8A,B,D). A 2 (RB vs. II) by 2 (Correct vs. Incorrect) ANOVA was performed on post-feedback mean amplitudes at 200–400 ms from a cluster of seven central electrodes (Figure 3D). The analysis yielded a main effect of accuracy (*F*_(1,11)_ = 43, *p* < 0.001, *η*_p_^2^ = 0.78), but no effect of distribution type (*F*_(1,11)_ = 0, *p* = 0.99, *η*_p_^2^ = 0), and no interaction between distribution type and accuracy (*F*_(1,11)_ = 0.25, *p* = 0.6, *η*_p_^2^ = 0.02).

However, because the P300 is frequently associated with expectancy violations (Polich, 2007) and is larger when participants receive unexpected feedback (Hajcak et al., 2005), we hypothesized that participants who were better at RB categorization would show lower P300 response to correct feedback than would participants who had less-developed rules. To test this idea, we correlated categorization accuracy with P300 amplitude to correct feedback signals. Confirming our hypothesis, we found that accuracy was inversely correlated with P300 amplitude for the RB distribution (Figure 8C; *r*_(11)_ = −0.71, *p* = 0.01), but not for the II distribution (Figure 8F; *r*_(11)_ = 0.07, *p* = 0.83).

Because the stimulus-locked LPC during categorization and the feedback-locked P300 both appear to index effective learning in the RB condition, but not in the II condition, we looked to see whether they were related across participants. The LPC correct/incorrect subtraction is negatively correlated with the feedback P300 correct/incorrect subtraction in the RB condition (*r* = −0.59, *p* = 0.03), but not in the II condition (*r* = −0.08, *p* = 0.82). We believe the negative correlation observed in RB trials indicates that better performers are generally more confident in their learning. Thus, they tend to update their memory more on correct than incorrect trials (larger Correct/Incorrect LPC difference) and also tend to be less surprised when they receive positive feedback, (smaller Correct/Incorrect Feedback P300 difference). This correlation is dramatically absent in II learning suggesting that even good II learners do not have explicit awareness of their learning.

#### Prelearning ERPs

Our critical comparisons during category learning were between correct and incorrect trials within either RB or II distributions, not across the two distributions. Yet, we took steps to ensure that differences were not due to the nature of the stimuli in the RB vs. II distributions. Accordingly, we analyzed ERPs from prelearning at the same latencies and scalp locations used in the categorization analyses for N1 and LPC. Neither N1 (*t*_(10)_ = 1.0, *p* = 0.34) nor LPC (*t*_(10)_ = 0.11, *p* = 0.91) differed between the two distributions, confirming that effects can be ascribed to learning rather than physical stimulus differences.

## Discussion

ERP measures differentiated RB and II category-learning processes from each other. During categorization, differences in neural activity were observed in an early, occipital N1 ERP component in the form of differential correct/incorrect activity patterns for RB and II conditions (Figure 6). N1 amplitudes in the II condition were more negative for correct than for incorrect trials, while a trend toward the opposite pattern was observed in the RB condition. At a later latency, LPC amplitudes during RB learning were larger for correct than for incorrect trials, whereas LPC amplitudes during II learning categorization were not modulated by success (Figure 7). In addition, a central P300 ERP to positive feedback was correlated with accuracy for the RB but not the II condition (Figure 8). Together, these differences in brain waves associated with category learning expand on related results from neuropsychological and fMRI studies. In addition, the current findings add neurocognitive information about the temporal order of processing, as discussed further below. Moreover, the lack of ERP differences for stimuli prior to learning makes it possible to rule out trivial physical stimulus factors. Accordingly, we attribute these ERP differences to the distinctive neurocognitive computations engaged during category learning and use.

RB processing is thought to depend on hypothesis testing, whereby a candidate rule is evaluated by comparing the representation of the stimulus in the current trial to that of a representation of a category threshold. This evaluation requires selective attention and working memory, likely implemented in PFC, as well as the ability to form enduring mental representations of the rule and the threshold, dependent on the hippocampus and MTL. In contrast, II learning may be likened to gaining category expertize with complex objects such as faces (Bentin et al., 1996) or Greebles (Rossion et al., 2002).

ERP results were consistent with both of these descriptions. The more positive potential for correct compared to incorrect RB trials late during each trial (Figure 7) is similar to positive potentials that have been found in many different tasks and variously referred to as the P3b, P600, or LPC. These positive potentials with broad parietal topographies have been associated with working memory (Kok, 2001; Polich, 2007) and episodic memory retrieval (Paller et al., 1988, 2009; Halgren et al., 1994; Fernández et al., 1999; Guillem et al., 1999). The LPC found here may reflect retrieval/updating of the categorization rule and some mental representation of the boundary condition, two functions consistent with the function of anatomical regions previously associated with the RB category-learning system (Filoteo et al., 2005; Seger and Cincotta, 2006; Nomura et al., 2007a; Seger et al., 2011; Nomura and Reber, 2012). Likewise, we only found these LPC differences when participants’ categorization response patterns suggested they are using a simple rule based on a single feature. Similarly, the magnitude of the Correct/Incorrect difference was positively correlated with individual participant categorization success.

LPC potentials were also apparent in the II condition, but there were no reliable differences between Correct and Incorrect trials, and the magnitude of the Correct/Incorrect difference was unrelated to individual participant categorization success. One possible explanation for the elevation of the LPC here is that the neural machinery responsible for the LPC is engaged during the II condition; however it is not responsible for successful categorization. This interpretation of the LPC is consistent with context-updating theory whereby information from an incoming stimulus results in revision of a maintained mental representation (Donchin, 1981). Given the gradual nature of feedback learning it is likely that participants are updating the mental representation of the boundary condition throughout successful RB learning. In contrast, when participants are relatively confident of the rule they are using, but uncertain about whether a given stimulus is an A or B they may not update (lower LPC). In the II condition they are constantly trying to update their rule and/or boundary condition, but this does not result in successful learning. In this interpretation the neural systems responsible for the LPC is engaged during II learning, but it’s output is likely inhibited (Ashby and Maddox, 2011) and thus not responsible for the final behavioral decisions. Nomura and Reber (2012) proposed that RB and II systems are both active and interact competitively during categorization with the DLPFC resolving this competition based on appraising confidence in both systems. Our LPC ERP is consistent with this proposal that the explicit category-learning system is engaged in both the RB and II tasks, but it is only effective in guiding optimal categorization performance in the RB condition.

We also observed an early occipital Correct/Incorrect difference wave (Figure 6). A prior visual category learning study also as associated with implicit category learning N1 ERP (Curran et al., 2002). The authors speculated that this ERP could be related to the N170 ERP frequently observed in studies of face processing (e.g., Bentin et al., 1996) and expert categorization (e.g., Tanaka and Curran, 2001; Rossion et al., 2002). This type of processing frequently engages extrastriate visual cortex (e.g., Kanwisher et al., 1997; Gauthier et al., 1999), an area found to be more active in the II condition of this task (Nomura and Reber, 2012) and previously implicated in several other category-learning tasks (Reber et al., 1998a,b). The early time-course of our effect suggests a shaping of visual perception that occurs as part of the category learning process in tasks like II categorization.

One hypothesis is that the observed N1 may reflect the degree to which a participant uses holistic processing to process the sine-wave gratings. Ashby and Maddox (2011) have argued that II tasks encourage participants to integrate perceptual information from different stimulus features at a predecisional level. In contrast, RB tasks encourage participants to consider single features and judge them against a rule.^1^ Thus, holistic processing is advantageous with the II distribution, while it may be detrimental with the RB distribution where attention to spatial orientation could distract the participant from focusing on the spatial frequency information necessary to appraise the rule used to define the RB categories in this study. The presence of the N1 effect in both RB and II conditions is also consistent with the idea that both processes are regularly active during categorization, but that the results of the earlier II process may be inhibited to allow the RB to respond (Ashby and Maddox, 2011).

The electrophysiological methods used in this study also allowed us to separate neural correlates of categorization accuracy from neural signals accompanying feedback. We observed a differential Correct/Incorrect P300 response during feedback that did not differ in amplitude between RB and II conditions (Figure 8). However, feedback-related P300 amplitude on correct trials negatively correlated with RB accuracy but not with II accuracy (Figures 8C,F). P300 responses to feedback may be sensitive to expectancies, as in prior studies with very different tasks (e.g., Courchesne et al., 1977; Duncan-Johnson and Donchin, 1977; Johnson and Donchin, 1980), and when participants receive unexpected feedback (Hajcak et al., 2005). In the present case, the observed correlations may reflect an explicit/implicit distinction between RB and II category-learning strategies. Specifically, over trials participants in the RB condition are developing a hypothesized categorization rule including a representation for the boundary condition for that rule. Each new stimulus is considered with respect to this context. When those expectations are confirmed by positive feedback, participants are less surprised the more confident they are in their rule and boundary condition representation. In contrast, while participants perform similarly with respect to accuracy in the II condition, they do not become confident in their rule because an explicit RB rule is not driving their performance. This result is consistent with participants’ self-reports, which indicate confidence in their rule description after RB learning and little to no confidence after II learning. Thus, these results provide further evidence for an explicit/implicit distinction between RB and II learning.

The majority of our ERP analyses in this study are based on correct/incorrect subtractions that seek to isolate what is unique about successful RB and II categorization. The advantage of this subtractive approach (see also Nomura et al., 2007a) is that aspects of the two tasks that may be common such as seeing the stimulus, making a response, and hearing feedback are subtracted away leaving us with what is unique. However, this means by definition that our descriptions of RB and II category learning are incomplete because these common processes are certainly part of the whole mechanism and may be important to achieve a full understanding of category learning. Likewise, it is difficult for us to use this approach to look at how the category-learning processes changes over time as so does the balance of correct and incorrect trials. Given successful learning, correct trials are more abundant at the end of the experiment than at the beginning when their neural correlates are likely more affected by guessing with either RB or II distributions. These factors are both important, particularly when we consider categories that may be learned and used frequently over the course of a lifetime. Recently, in their ambitious study of expertise in category learning (participants performed 10,000 trials over the course of the experiment compared to our 320 trials), Waldschmidt and Ashby (2011) demonstrated that even when considering just a single distribution type the neural correlates responsible for category use can change as participants approach expertise in categorization.

In summary, the present ERP findings illustrate two distinct neurocognitive processes responsible for successful category learning. These processes appear to compete on each categorization trial. The II process utilizes a network including, but not limited to the occipital cortex likely reflecting changes in perceptual processing as a result of implicit category learning. In contrast the more deliberative RB process occurs later during processing of a stimulus and employs more anterior cortical regions associated with working and long-term memory, most likely in association with MTL networks. In addition, neural activity measured during feedback suggests participants are aware of their learning when using an RB process to make their categorization decisions, but not when they are using the II process. Our findings do not appear to arise from differences in stimuli, but rather stem from differences in the neurocognitive processes which can be engaged while learning different types of categories. This experimental approach provides new perspectives on these category-learning mechanisms as well as a new way to investigate their interaction and competition during learning.

### Human Research Statement

Humans participated in this experiment according to procedures approved by the Northwestern University Institutional Review Board. Before beginning the experiment, participants were required to read and sign the informed consent form. They were encouraged to ask any questions and had the option of leaving at any time with no adverse consequences. The informed consent forms are kept on record in the lab.

## Conflict of Interest Statement

The authors declare that the research was conducted in the absence of any commercial or financial relationships that could be construed as a potential conflict of interest.

## Footnotes

^1^While not used in this study, RB tasks can also require use of a conjunctive rule whereby information about more than one feature is evaluated against a more complex rule at a later stage of processing.
